# A PIP-mediated osmotic stress signaling cascade plays a positive role in the salt tolerance of sugarcane

**DOI:** 10.1186/s12870-021-03369-9

**Published:** 2021-12-13

**Authors:** Hanchen Tang, Qing Yu, Zhu Li, Feng Liu, Weihua Su, Chang Zhang, Hui Ling, Jun Luo, Yachun Su, Youxiong Que

**Affiliations:** 1 Key Laboratory of Sugarcane Biology and Genetic Breeding, Ministry of Agriculture and Rural Affairs, Fujian Agriculture and Forestry University, Fuzhou, 350002 Fujian China; 2 Key Laboratory of Pathogenic Fungi and Mycotoxins of Fujian Province (Fujian Agriculture and Forestry University), Fuzhou, 350002 Fujian China; 3 Key Laboratory of Genetics, Breeding and Multiple Utilization of Crops, Ministry of Education, College of Agriculture, Fujian Agriculture and Forestry University, Fuzhou, 350002 Fujian China; 4 College of Agriculture, Yulin Normal University, Yulin, 537000 Guangxi China

**Keywords:** Sugarcane, Aquaporin, Abiotic stress, Genetic transformation, Salinity tolerance

## Abstract

**Background:**

Plasma membrane intrinsic proteins (PIPs) are plant channel proteins involved in water deficit and salinity tolerance. PIPs play a major role in plant cell water balance and responses to salt stress. Although sugarcane is prone to high salt stress, there is no report on PIPs in sugarcane.

**Results:**

In the present study, eight PIP family genes, termed *ScPIP1–1*, *ScPIP1–2*, *ScPIP1–3*, *ScPIP1–4*, *ScPIP2–1*, *ScPIP2–2*, *ScPIP2–4* and *ScPIP2–5*, were obtained based on the sugarcane transcriptome database. Then, *ScPIP2–1* in sugarcane was cloned and characterized. Confocal microscopy observation indicated that ScPIP2–1 was located in the plasma membrane and cytoplasm. A yeast two-hybridization experiment revealed that ScPIP2–1 does not have transcriptional activity. Real time quantitative PCR (RT-qPCR) analysis showed that *ScPIP2–1* was mainly expressed in the leaf, root and bud, and its expression levels in both below- and aboveground tissues of ROC22 were up-regulated by abscisic acid (ABA), polyethylene glycol (PEG) 6000 and sodium chloride (NaCl) stresses. The chlorophyll content and ion leakage measurement suggested that *ScPIP2–1* played a significant role in salt stress resistance in *Nicotiana benthamiana* through the transient expression test. Overexpression of *ScPIP2–1* in *Arabidopsis thaliana* proved that this gene enhanced the salt tolerance of transgenic plants at the phenotypic (healthier state, more stable relative water content and longer root length), physiologic (more stable ion leakage, lower malondialdehyde content, higher proline content and superoxide dismutase activity) and molecular levels (higher expression levels of *AtKIN2*, *AtP5CS1*, *AtP5CS2*, *AtDREB2*, *AtRD29A*, *AtNHX1*, *AtSOS1* and *AtHKT1* genes and a lower expression level of the *AtTRX5* gene).

**Conclusions:**

This study revealed that the *ScPIP2–1*-mediated osmotic stress signaling cascade played a positive role in plant response to salt stress.

**Supplementary Information:**

The online version contains supplementary material available at 10.1186/s12870-021-03369-9.

## Background

Plants suffer from various environmental stresses during the process of growth and development. Salt, drought and alkali stresses are the dominant abiotic stresses in plants and can impede plant growth and development and thus production [1]. The water transport channel is one of the important defense components for resisting salt, drought and alkali stresses [2]. Regarding the water transport channel, there are three kinds of pathways: transmembrane, symplastic and apoplastic [3]. The former two accomplish water transport by cell-to-cell pathways or through plasmodesmata and membranes, and the last one transports water by water flow in the xylem [4]. However, the water transport channels could be blocked by casparian bands and suberin lamellae [3].

Aquaporins (AQPs) belong to the major intrinsic protein (MIP) family [5]. AQPs play a core role in water transport channels and are able to guide water transport across membranes between the protoplasm [6–10]. In plants, the molecular weights of AQPs are 23–30 kDa [5]. There are five water rings and six transmembrane α-helices in AQPs [11, 12]. According to subcellular localization and sequence similarities, AQPs in higher plants can be divided into five subfamilies: plasma membrane intrinsic proteins (PIPs), tonoplast membrane intrinsic proteins (TIPs), nodulin26-like major intrinsic proteins (NIPs), small and basic intrinsic proteins (SIPs) and X intrinsic proteins (XIPs) [13, 14]. Based on the N-terminal length of the proteins, PIPs are divided into PIP1s and PIP2s, which have different roles in plants when they respond to various stresses [5, 6, 15].

*AQP* genes have been cloned and characterized in several plant species, such as *Arabidopsis thaliana* [11], *Oryza sativa* [16], *Zea mays* [17] and *Solanum lycopersicum* [18]. As reported, *AQPs* play a major role during plant responses to water stress [6]. The expression of *AQPs* maintains the balance of water when plants suffer from various abiotic stresses such as salt, drought and cold [5]. PIPs are regarded as the major water transport proteins in roots and leaves of higher plants [5]. Previous research revealed that different *PIP* family members had various expression patterns and functions when plants were exposed to osmotic stress [19, 20]. When *Arabidopsis* was treated with 250 mmol·L^− 1^ mannitol for 2 d, the expression levels of../%25E5%258A%259E%25E5%258,525 AC/360/Youdao/Dict/8.5.2.0/resultui/html/index.html - /javascript:; *AtPIP1–3*, *AtPIP1–4*, *AtPIP2–1* and *AtPIP2–5* were 5 times as high as those in control leaves, but those of *AtPIP1–5*, *AtPIP2–2*, *AtPIP2–3* and *AtPIP2–6* decreased by 10% compared with the control [20]. Moreover, the expression levels of the *PIP2* gene in *Arabidopsis* roots declined in response to salt stress [21]. The association between the health status of *Arabidopsis* and the higher expression levels of *PIPs* (*AtPIP1–4* and *AtPIP2–5*) showed that the expression and activity of *AtPIP1–4* and *AtPIP2–5* could regulate water balance in plants after osmotic stress [22]. During the process of drought stress, the expressions of *NtPIP1–1* and *NtPIP2–1* were down-regulated in *Nicotiana benthamiana* [23]. Moderate expression levels of *OsPIP1–1* can increase the seed yield, salt resistance, root hydraulic conductivity and seed germination rate of rice [24]. Furthermore, the expressions of *OsPIP2* genes were positively correlated with the level of salt stress, and this phenomenon was similar to drought stress [25, 26]. In *Rosa rugosa*, RhPTM (PM-tethered MYB) could interact with RhPIP2–1 to maintain the water balance in cells [27]. These reports confirmed that *PIPs* could aid the response to salt stress in plants such as *Z. mays* [17], *O. sativa* [16] and *A. thaliana* [11]. However, there is no report on the identification and characterization of *PIP* genes in sugarcane (*Saccharum* spp. hybrid, var. ROC22) until now.

Sugarcane, the most important sugar crop in the world, is often planted in tropical and subtropical regions. Salt stress hinders sugarcane growth and development and also reduces its production. In the present study, first, according to the transcriptome database, eight sugarcane *PIP* genes (named *ScPIPs*) were obtained and analyzed by the bioinformatics method. Second, a full-length cDNA sequence of *ScPIP2–1*, homologous to the rice salt-resistant gene *OsPIP2–1* (AF062393.1) [26], was cloned from the main sugarcane variety ROC22 (*Saccharum* spp. hybrid) in mainland China. The amino acid sequence, subcellular location and transcriptional activity of the ScPIP2–1 protein were analyzed. Third, in order to understand the expression patterns of *ScPIP2–1* in ROC22, tissue-specific expression and the expression of *ScPIP2–1* under polyethylene glycol (PEG) 6000, sodium chloride (NaCl) and abscisic acid (ABA) stresses were detected. Finally, the biological functions of *ScPIP2–1* under salt stress were also investigated by transient overexpression of *ScPIP2–1* in *N. benthamiana* leaves combined with stable overexpression in *A. thaliana*. The above results suggested that *ScPIP2–1* might enhance salt tolerance in plants mainly by maintaining cell homeostasis and by eliminating reactive oxygen species (ROS). In other words, the salt tolerance of transgenic *Arabidopsis* could be enhanced by the *ScPIP2–1*-mediated osmotic stress signaling cascade.

## Results

### Identification and classification of sugarcane PIP family genes

**Fig. 1:**
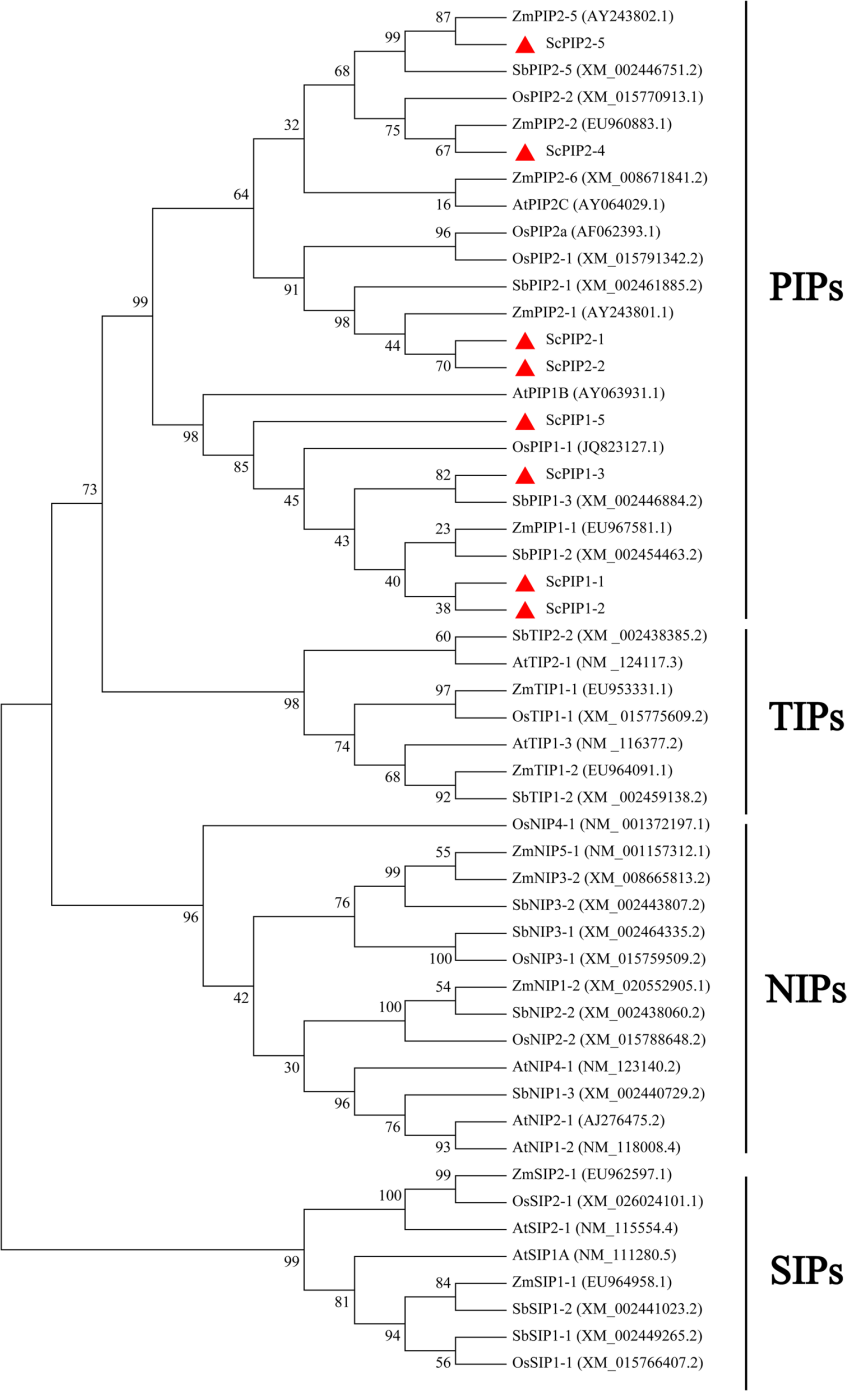
The phylogenetic tree of sugarcane and other plant AQPs. The red filled triangle represents the proteins acquired from sugarcane in this study. The GenBank accession numbers follow the protein names. At, *Arabidopsis thaliana*; Sb, *Sorghum bicolor*; Zm, *Zea mays*; Os, *Oryza sativa*

Based on our previous sugarcane transcriptome, eight ScPIP family genes, namely, *ScPIP1–1* (MZ362000), *ScPIP1–2* (MZ362001), *ScPIP1–3* (MZ362002), *ScPIP1–4* (MZ362003), *ScPIP2–1* (MZ362004), *ScPIP2–2* (MZ362005), *ScPIP2–4* (MZ362006) and *ScPIP2–5* (MZ362007), were mined (Table S1). Bioinformatics analysis showed that these eight ScPIP proteins had a relative molecular mass of 30–31 kDa, isoelectric points (*p*Is) of 6.64–9.00, and all belonged to stable hydrophobins (Table S2). A phylogenetic tree showed that these ScPIP proteins and AQP proteins from other higher plants were divided into four subfamilies: PIPs, TIPs, NIPs and SIPs. All eight ScPIP proteins belonged to the PIP subfamily (Fig. 1).

### Cloning and sequence analysis of the *ScPIP2–1* gene

**Fig. 2:**
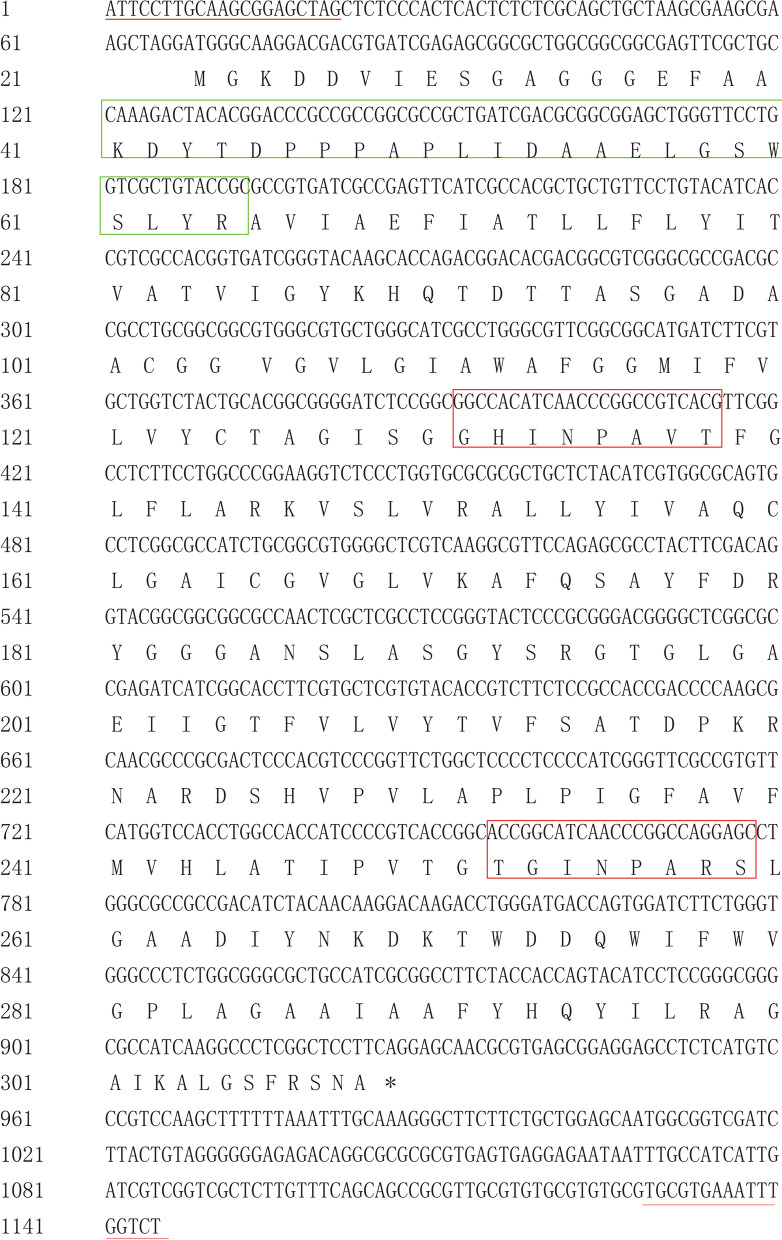
Nucleotide acid sequences and deduced amino acid sequences of the *ScPIP2–1* gene obtained by RT-PCR. The sequences of the NPA motifs are highlighted with red frames, PIP characteristic sequences are highlighted with green frames, and the primers for RT-PCR are highlighted with red underlining. *, stop codon

**Fig. 3:**
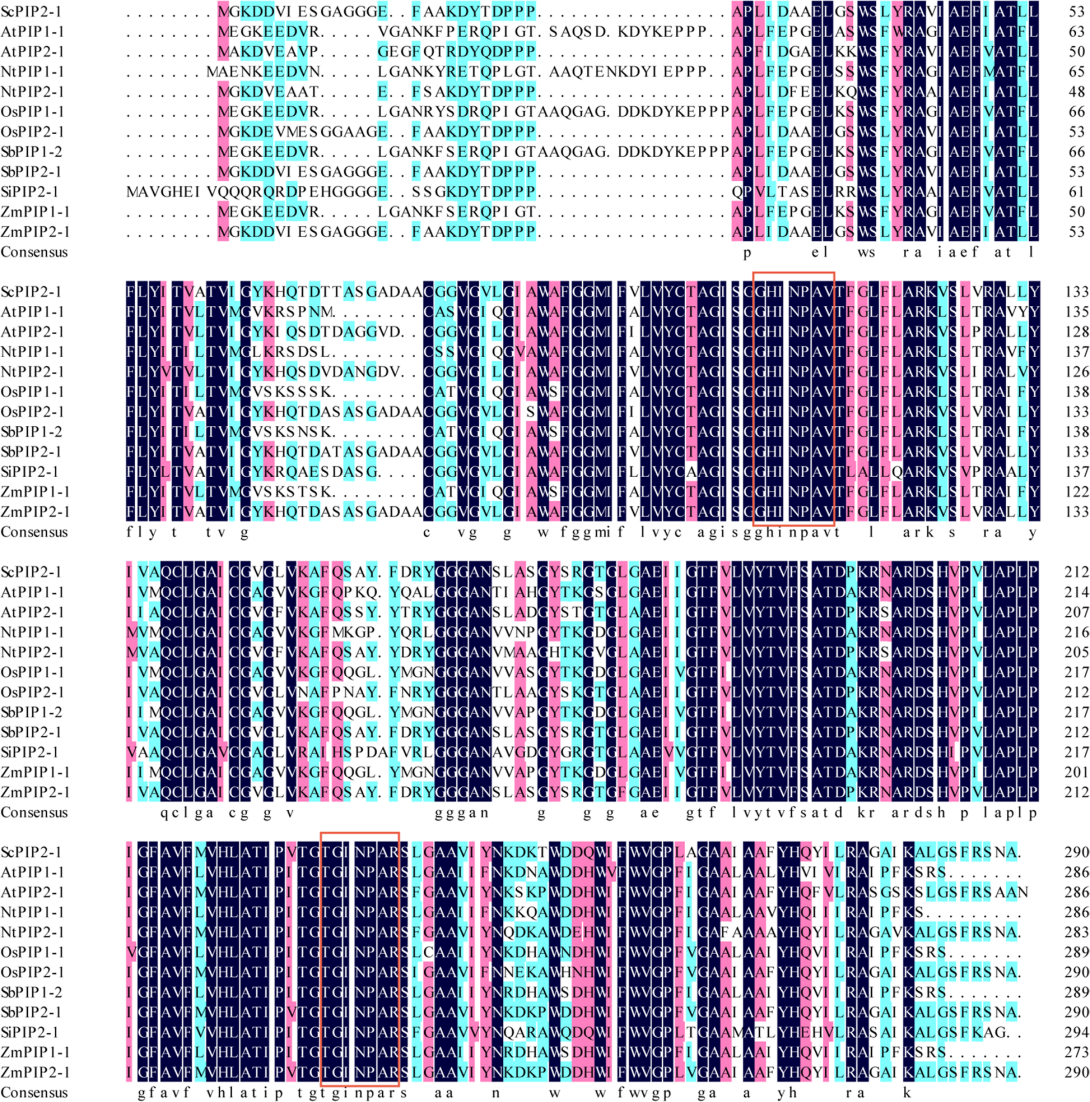
Amino acid sequence alignment of ScPIP2–1 and PIPs from other plant species based on DNAMAN (version 6.0.3.99, Lynnon Biosoft) software. AtPIP1–1, AY063931.1; AtPIP2–1, NM_115202.3; NtPIP1–1, AF440271.1; NtPIP2–1, NM_001326279.1; OsPIP2–1, AF062393.1; OsPIP1–1, NM_001054207; SbPIP1–2, XM_002454463.2; SbPIP2–1, XM_002461885.2; SiPIP2–1, XM_004986439.2; ZmPIP2–1, AY243801.1; ZmPIP1–1, EU967581.1. The sequences of the NPA motifs are highlighted by red rectangles

A full-length cDNA sequence of *ScPIP2–1* was cloned from the 24-h 250 mmol·L^− 1^ NaCl-treated ROC22 leaves by reverse transcription-polymerase chain reaction (RT-PCR). The cDNA length of *ScPIP2–1* was 1145 bp, including an open reading frame (ORF) of 873 bp that encoded 290 amino acids (Fig. 2). Moreover, the ScPIP2–1 protein had the MIP conservative structure of the NPA-motif (Asn-Pro-Ala) and the PIP feature structures (P/KDYXE/DPPP/RX3-4E/DXXELXXWSFY/WR, and GGGANXXXXGY) (Jang et al., 2004). Sequence alignment indicated that ScPIP2–1 was highly homologous with PIPs in *Arabidopsis*, rice, tobacco, maize and sorghum. However, the N-terminal amino acid sequences of these homologous proteins are quite different (Fig. 3).

### Subcellular localization analysis of the ScPIP2–1 protein

**Fig. 4:**
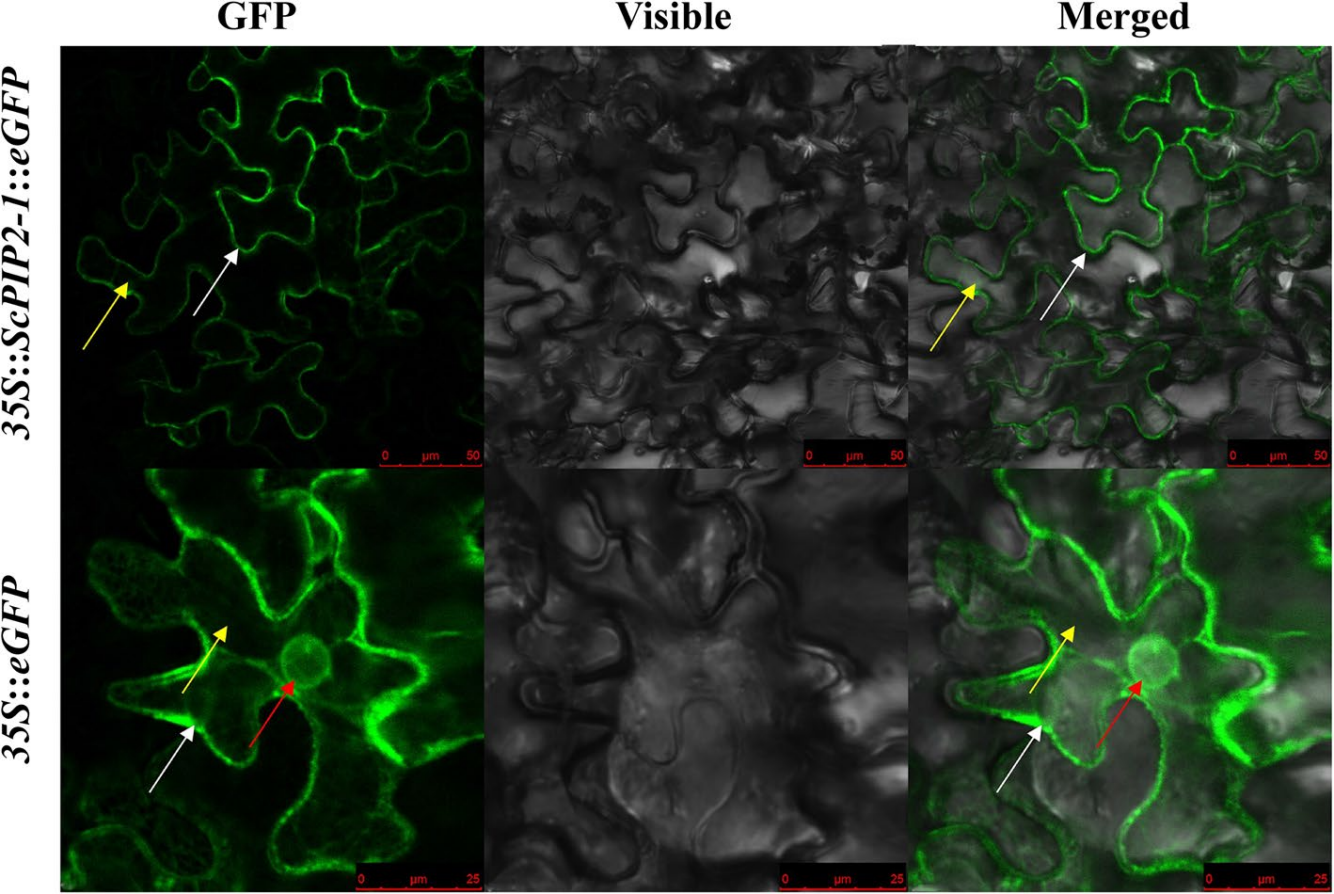
Subcellular localization of ScPIP2–1 and empty vector in *Nicotiana benthamiana* leaves after 2 days of infiltration. Image of epidermal cell captured using green fluorescence, merged light and visible light. Red arrows mark the cell nucleus, yellow arrows mark the cytoplasm, and white arrows mark the plasma membrane

Subcellular localization analysis (Fig. 4) showed that *35S*::*ScPIP2–1*::*eGFP* was expressed in the plasma membrane and cytoplasm, and *35S*::*eGFP* was expressed in the plasma membrane, cell nucleus and cytoplasm.

### Transcription activation activity of the ScPIP2–1 protein

**Fig. 5:**
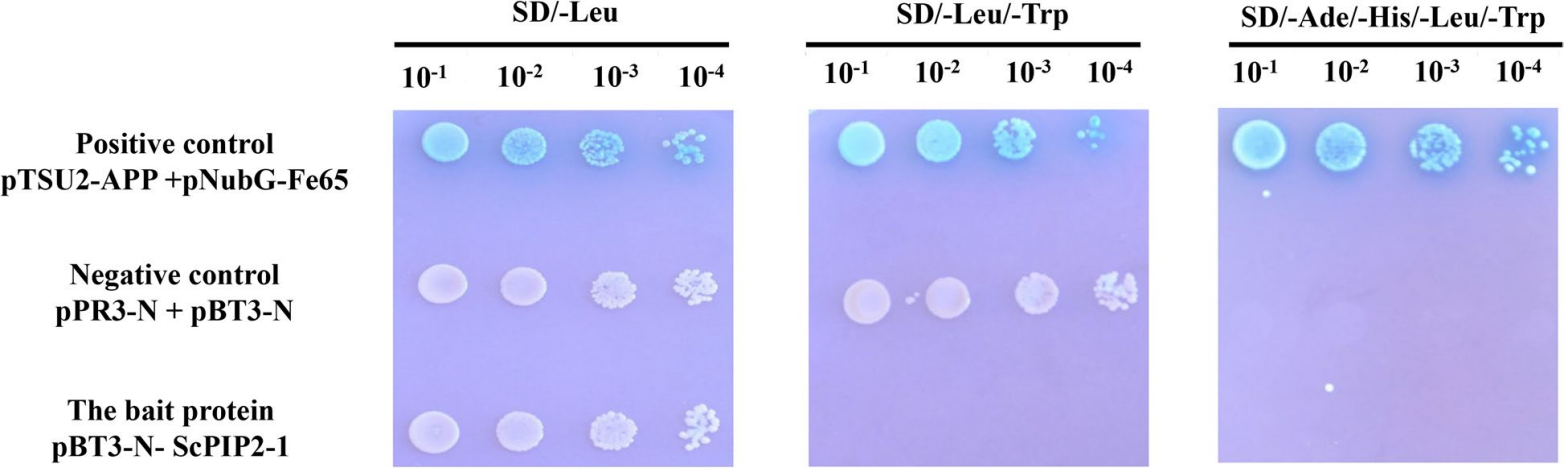
Test of the ScPIP2–1 trans-activation assay. The media were added with X-Gal (5-bromo-4-chloro-3-indoxyl-D-galactopyranoside). 10^− 1^, 10^− 2^, 10^− 3^ and 10^− 4^ represent the sample concentrations diluted ten-fold, hundred-fold, thousand-fold and ten thousand-fold, respectively. SD/−Leu, the synthetic dropout medium without leucine; SD/−Leu/−Trp, the synthetic dropout medium without leucine and tryptophan; SD/−Ade/−His/−Leu/−Trp, the synthetic dropout medium without adenine, histidine, leucine and tryptophan

The DUAL membrane yeast two-hybrid system was used to detect the transcriptional activity of ScPIP2–1. Compared with the positive and negative control, pBT3-N-*ScPIP2–1* did not possess transcriptional activity and showed no toxicity to the yeast strain NMY51 (Fig. 5).

### Tissue-specific expression of the *ScPIP2–1* in different sugarcane tissues

**Fig. 6:**
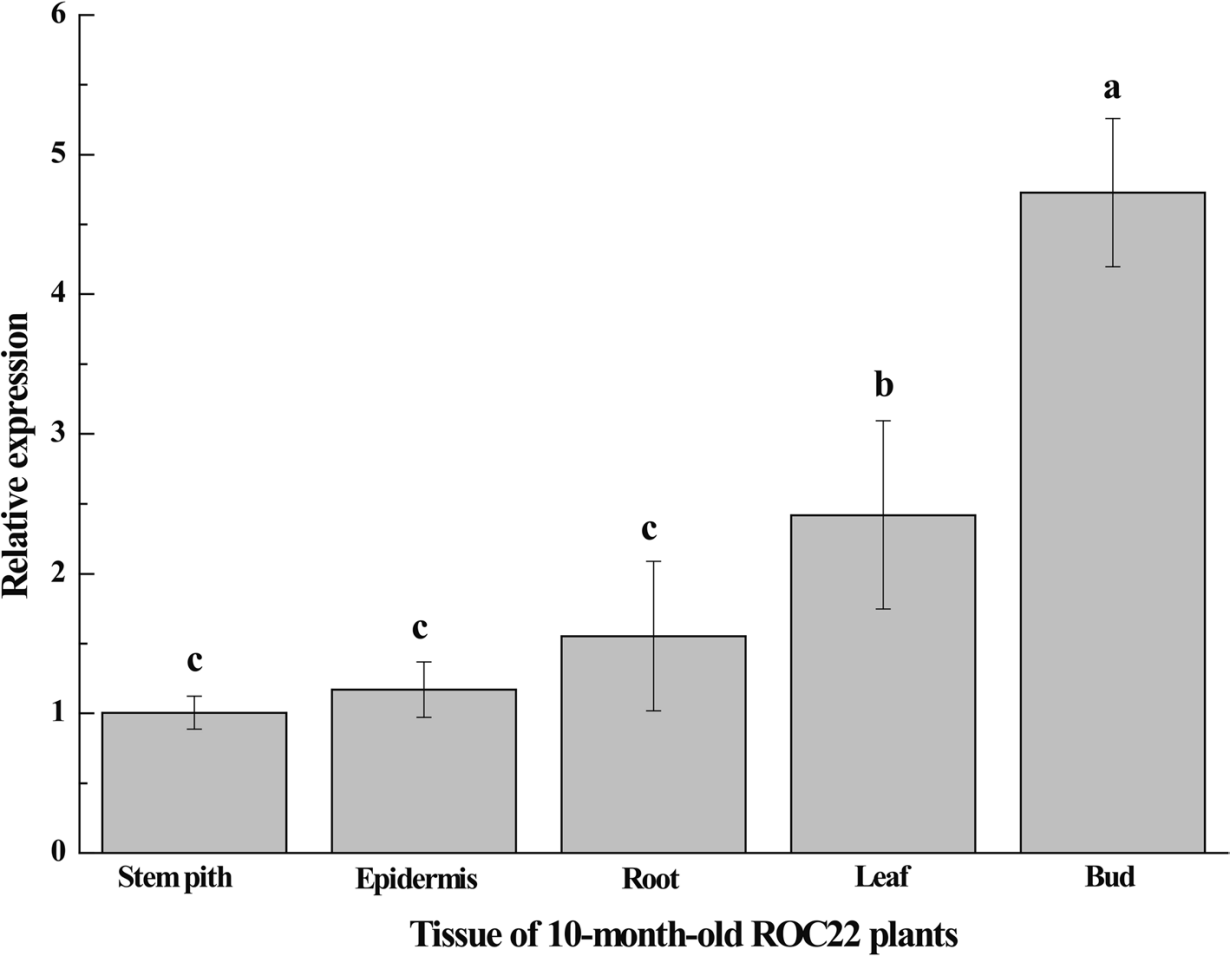
Tissue-specific expression analysis of the *ScPIP2–1* gene in 10-month-old sugarcane ROC22 plants. Data were normalized to the expression level of the glyceraldehyde-3-phosphate dehydrogenase (*GAPDH*) gene. All data are expressed as means ± standard error (*n* = 3). Different lowercase letters indicate a significant difference (*P* < 0.05) compared to the control, as determined by Duncan’s test

Real time quantitative PCR (RT-qPCR) was performed to determine the expression patterns of the *ScPIP2–1* gene in different sugarcane tissues, including the stem pith, epidermis, root, leaf and bud. As shown in Fig. 6, the *ScPIP2–1* gene was constitutively expressed in all these sugarcane tissues but mainly in the bud, leaf and root, which was 4.71-, 2.41- and 1.55-fold higher, respectively, than that in the stem pith.

### Expression of the *ScPIP2–1* gene in response to various abiotic stresses

**Fig. 7:**
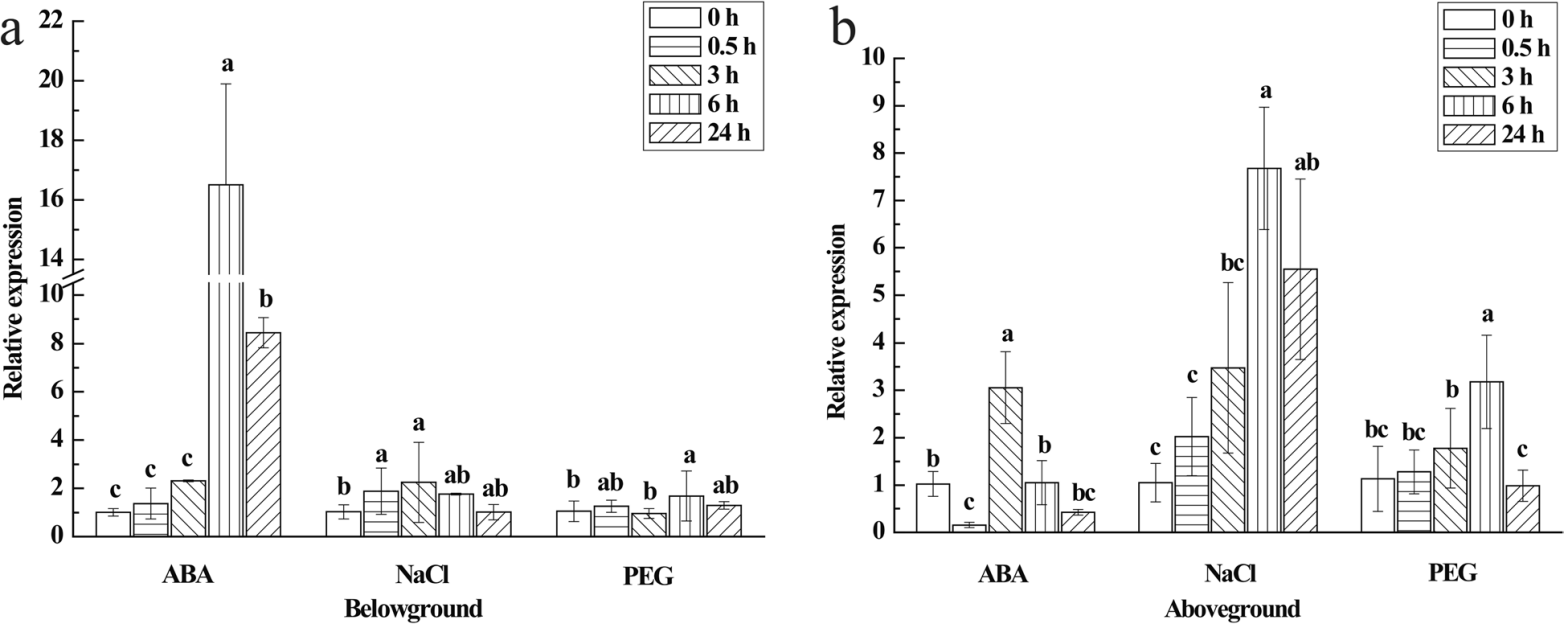
Relative expression of *ScPIP2–1* after the application of exogenous plant hormones and abiotic stress. **a** The results of belowground tissues of ROC22. **b** The results of aboveground tissues of ROC22. *ScPIP2–1* transcript abundance in ROC22 plants was assessed in the presence of 0.1 mmol·L^− 1^ ABA, 250 mmol·L^− 1^ NaCl and 25% PEG 6000. The data were normalized to the expression level of the glyceraldehyde-3-phosphate dehydrogenase (*GAPDH*) gene. All data are expressed as means ± standard error (*n* = 3). Different lowercase letters indicate a significant difference (*P* < 0.05), as determined with Duncan’s test. ABA, abscisic acid; NaCl, sodium chloride; PEG 6000, polyethylene glycol 6000

In the case of ABA, NaCl and PEG stresses, except for the downregulation in aboveground tissues at 0.5 h under ABA treatment, the expression levels of the *ScPIP2–1* gene in both below- and aboveground tissues of ROC22 were up-regulated (Fig. 7). During ABA stress, the *ScPIP2–1* gene exhibited a sensitive response. Its expression levels peaked in belowground tissues at 6 h and in aboveground tissues at 3 h, which were 16.36- and 2.99-fold higher, respectively, than those of the control. In response to NaCl, the expression levels of the *ScPIP2–1* gene were increased from 0.5 h to 3 h in belowground tissues and from 0 h to 6 h in aboveground tissues but remained unchanged at the other time points. In response to PEG 6000, the expression levels of the *ScPIP2–1* gene increased at 6 h in both below- and aboveground tissues of ROC22, which were 1.59- and 2.81-fold higher, respectively, than those of the control. However, the expression levels of *ScPIP2–1* remained stable at the other time points of PEG 6000 treatment. These results suggested that *ScPIP2–1* had a positive response to salt and PEG 6000 stresses, most probably mediated by the ABA signaling pathway.

### Transient overexpression of *ScPIP2–1* gene regulated the responses of *N. benthamiana* leaves to salt stress

**Fig. 8:**
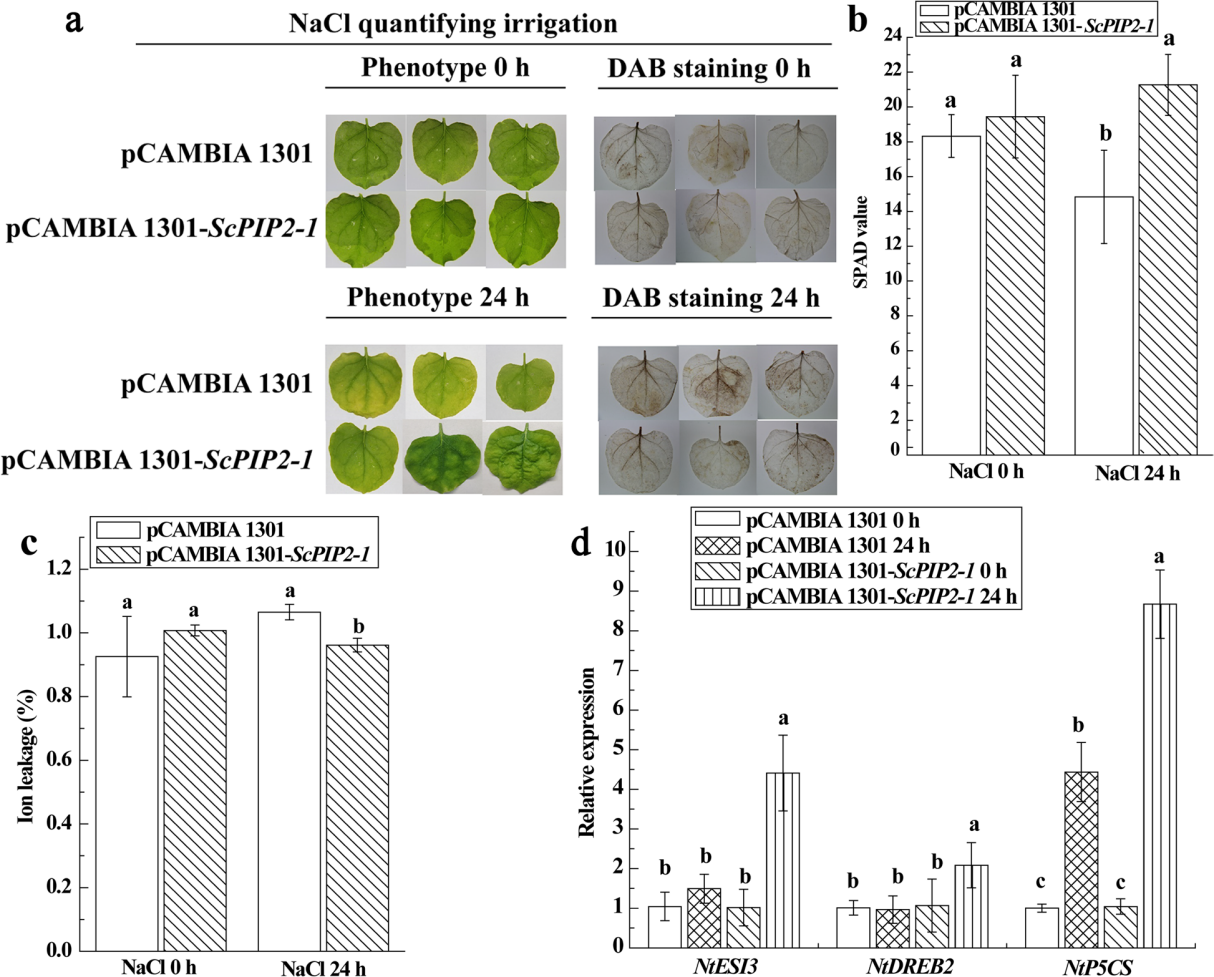
Effect of transient overexpression of the *ScPIP2–1* gene in *Nicotiana benthamiana* leaves. **a** The chlorosis and DAB staining of *N. benthamiana* leaves after 24-h salt stress. **b** and **c** SPAD value and ion leakage in the *N. benthamiana* leaves after 24-h salt stress. SPAD represents the amount of chlorophyll, and ion leakage represents the value of conductivity. **d** Expression of three tobacco osmotic stress-related marker genes in the *N. benthamiana* leaves after 24-h salt stress. The *NtEF1-α* gene was used for data normalization. All data points are presented as means ± standard error (*n* = 3). Different lowercase letters indicate a significant difference, as determined with Duncan’s test (*P* < 0.05)

After transient overexpression of *ScPIP2–1* in *N. benthamiana* leaves for 2 days, the overexpression tobacco plants were treated with 250 mmol·L^− 1^ NaCl for 24 h. Then, the salt tolerance of tobacco leaves was determined at the physiologic, phenotypic and molecular levels (Fig. 8). As shown in Fig. 8a, a darker DAB staining color was observed in the control (pCAMBIA 1301) than in *ScPIP2–1* leaves. Moreover, the phenomenon of stronger salt tolerance was demonstrated by a higher SPAD index and more stable ion leakage in *ScPIP2–1* overexpression plants after 24-h salt treatment (Figs. 8b, c). According to the results of Fig. 8d, the expression levels of salt-associated marker genes (*NtESI3*, *NtDREB2* and *NtP5CS*) in *ScPIP2–1* leaves were increased, which were 4.32-, 1.95- and 8.30-fold higher, respectively, than those of the control. These results laid the foundation for genetic transformation experiments and provided evidence for the *ScPIP2–1* gene to respond to salt stress.

### Overexpression of *ScPIP2–1* gene improved the salt stress tolerance in transgenic *Arabidopsis*

**Fig. 9:**
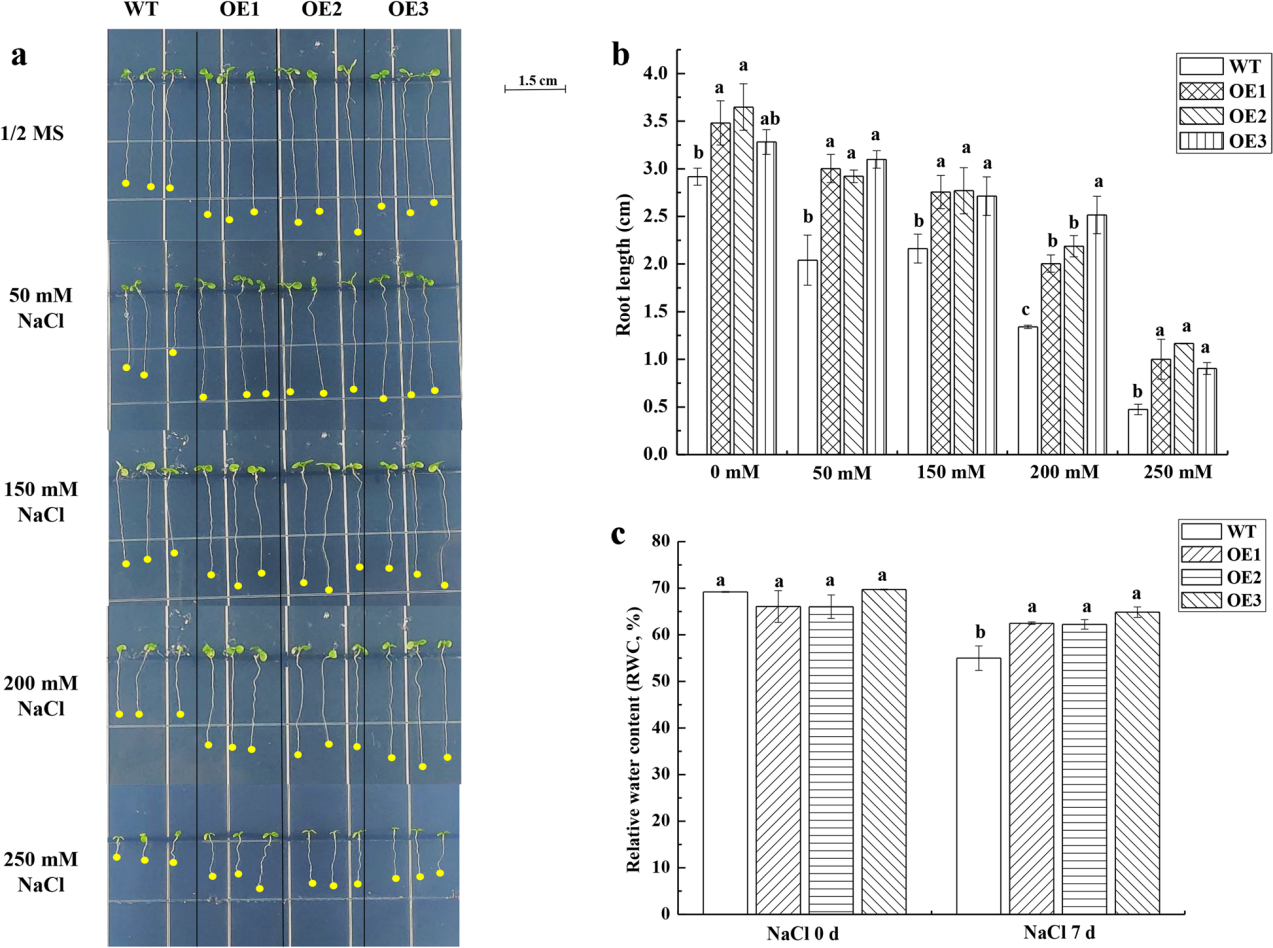
Root length of T_3_ *ScPIP2–1-*transgenic *Arabidopsis* seedlings at different NaCl concentrations. **a** Observation of root length under different concentrations of sodium chloride (NaCl). **b** Measurement of root length under different concentrations of NaCl by Image J 1.8.0. **c** Relative water content (RWC) of *Arabidopsis* plantlets under treatment of 250 mmol·L^− 1^ NaCl. Bars with different superscripts differ significantly (*P* < 0.05), and error bars represent the standard error of each treatment group (*n* = 3). WT, wild-type *Arabidopsis*; OE1, OE2 and OE3, three different lines of transgenic *Arabidopsis*

**Fig. 10:**
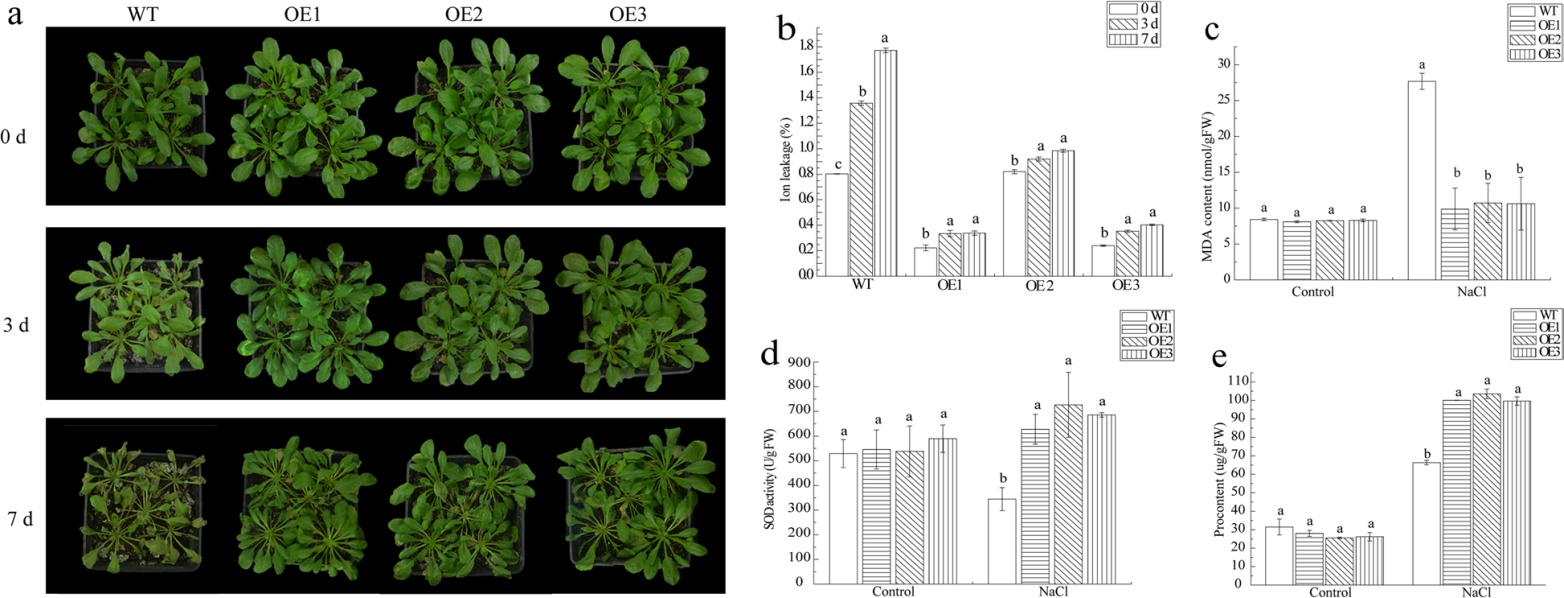
Detection of phenotypic and physiological indexes of T_3_ transgenic *Arabidopsis* under salt stress. **a** The growth status of transgenic and WT plants during 250 mmmol·L^− 1^ sodium chloride (NaCl) treatment. **b** Ion leakage. **c** The content of malondialdehyde (MDA). **d** The activity of superoxide dismutase (SOD). **e** The content of proline (Pro). Bars with different superscripts differ significantly (*P* < 0.05), and error bars represent the standard error of each treatment group (*n* = 3). WT, wild-type *Arabidopsis*; OE1, OE2 and OE3, three different lines of transgenic *Arabidopsis*

Here, a functional analysis of *Arabidopsis* overexpression *ScPIP2–1* for salt tolerance was conducted, and the indexes were measured at phenotypic, physiological and molecular levels. Three lines of homozygous transgenic *Arabidopsis* (OE1, OE2 and OE3) were selected in 1/2 MS medium containing hygromycin (HYG). At the phenotypic level, the transgenic *Arabidopsis* seedling had longer root length than wild-type *Arabidopsis* (WT) under 0–250 mmol·L^− 1^ NaCl (Fig. 9). In addition, compared to the 5-week-old transgenic *Arabidopsis*, the leaves of WT turned yellow and its growth was inhibited under 250 mmol·L^− 1^ NaCl stress (Fig. 10a). These results indicated that transgenic *Arabidopsis* might have better water absorption ability. As shown in Fig. 10c, the transgenic plantlets had a significantly higher relative water content (RWC) than WT plantlets after 250 mmol·L^− 1^ NaCl treatment.

**Fig. 11:**
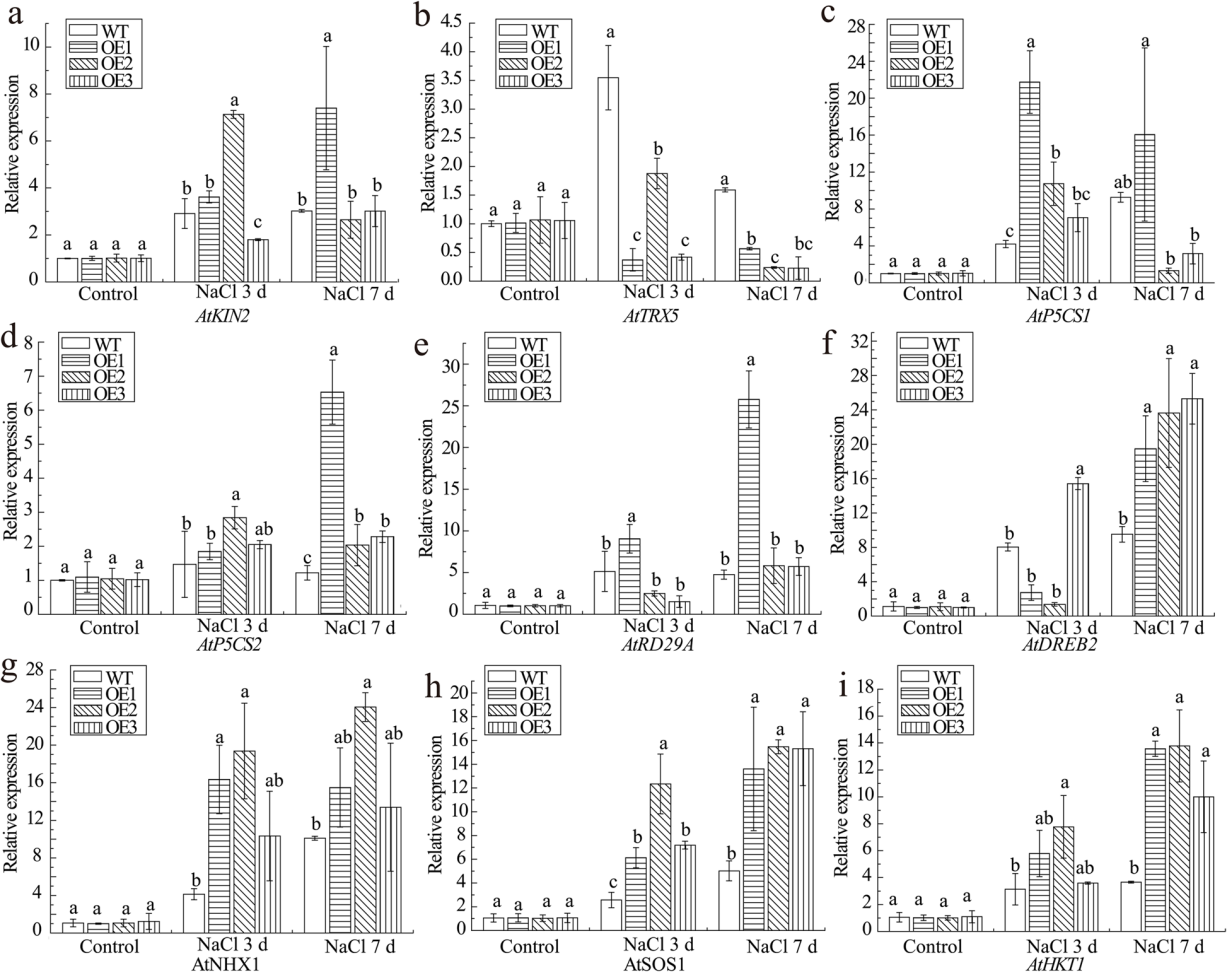
Expression of salt stress-related genes in *ScPIP2–1-*transgenic *Arabidopsis* based on RT-qPCR. *AtKIN2*, *AtDREB2* and *AtRD29A* represent the osmotic stress-defense genes. *AtTRX5* represents the reactive oxygen species (ROS) elimination gene. *AtP5CS1* and *AtP5CS2* represent the proline (Pro) synthesis-related genes. *AtNHX1*, *AtSOS1* and *AtHKT1* represent the ion exchange-correlated genes. *Arabidopsis* was grown in soil and was treated with 250 mmol·L^− 1^ sodium chloride (NaCl). Bars with different superscripts differ significantly (*P* < 0.05), and error bars represent the standard error of each treatment group (*n* = 3). WT, wild-type *Arabidopsis*; OE1, OE2 and OE3, three different lines of transgenic *Arabidopsis*

As Figs. 10b–e and Fig. 11 show, the transgenic *Arabidopsis* exhibited a stronger salt tolerance at the physiological and molecular levels. It was noted that the transgenic *Arabidopsis* had a higher proline (Pro) content, and the expression levels of Pro synthesis-related genes *AtP5CS1* and *AtP5CS2* were up-regulated by salt stress (Fig. 10e and Figs. 11c, d). According to the results of Fig. 10b and Figs. 11a, e and f, the transgenic *Arabidopsis* had a more stable intracellular environment than WT, which was exhibited by the insignificantly increased ion leakage (3–7 days) and the higher expression levels of osmotic stress-defense genes *AtKIN2*, *AtDREB2* and *AtRD29A*. Three genes, *AtNHX1*, *AtSOS1* and *AtHKT1*, which correlated with ion transport, were all up-regulated after 250 mmol·L^− 1^ NaCl treatment (Figs. 11g, h, i). These phenomena confirmed that overexpression of *ScPIP2–1* could enhance ion exchange to improve the salt tolerance of transgenic plants. Regarding the correlation between ROS removal and salt tolerance, the transgenic *Arabidopsis* had less malondialdehyde (MDA) and stronger superoxide dismutase (SOD) activity than WT, and the expression levels of the *AtTRX5* gene, which is related to ROS removal, were down-regulated, suggesting that overexpression of *ScPIP2–1* could restrict *AtTRX5* expression by taking part in ROS elimination (Figs. 10c, d and Fig. 11b).

## Discussion

AQPs play important roles as water channels in regulating water balance [6–10]. At present, many AQPs from various plant species have been used in transgenic research to improve salt stress tolerance in plants [28–31]. In this study, eight AQPs, all of which belong to the PIP subfamily, were mined from the sugarcane transcriptome database (Fig. 1). Johanson et al. suggested that the naming of AQPs should refer to the protein subcellular localization and sequence homology [11]. In subsequent research, the AQP proteins located in the plasma membrane and cytoplasm were renamed as PIPs in higher plants [5, 11]. Our research revealed that ScPIP2–1 was localized in the plasma membrane and cytoplasm (Fig. 4), which is in accordance with previous studies [27, 29]. Protein-protein interactions play an important role in plant development and growth [32]. Based on previous research in roses, RhPTM could interact with RhPIP2–1, indicating its important role in maintaining water balance [27]. In the present study, pBT3-N-ScPIP2–1 did not possess transcriptional activity and showed no toxicity to the yeast strain NMY51 (Fig. 5), suggesting that it could be used for yeast two-hybrid screening in future research.

Temporal and spatial expression of AQPs often represents their diverse functions [9]. *SbAQPs* were mainly expressed in roots and played major roles in sorghum under waterlogging [33]. *AtPIP1–4* and *AtPIP2–5* were mainly expressed in the leaf and root when *Arabidopsis* was exposed to salt and drought stresses [22]. Likewise, the tissue-specific expression of *ScPIP2–1* (Fig. 6) proved that it may play an important role in maintaining water transport in leaf and root tissues. Accordingly, we speculated that *ScPIP2–1* also participated in the sugarcane cell development due to the fact that *ScPIP2–1* was mainly expressed in bud tissues of sugarcane (Fig. 6), which is similar to the phenomenon that *RhPIP2–1* participated in rose petal cell swelling with higher expression in the meristem [34]. ABA is a major phytohormone that can enhance the tolerance of osmotic stress in plants [35]. In this study, the expression levels of the *ScPIP2–1* gene in both below- and aboveground tissues of ROC22 were increased by ABA treatment (Fig. 7). The transcripts of the *ScPIP2–1* gene were also up-regulated under NaCl and PEG 6000 stresses (Fig. 7). The above phenomena were the same as in many previous studies, for example, the expression levels of *ClPIP* were up-regulated under ABA treatment and had a positive response to osmotic stresses in *Citrullus lanatus* [36], and *PvPIP2–9* withstood salt and drought stresses through ABA signal regulation in *Panicum virgatum* [37]. Water deficit can cause difficulty in root water absorption, mainly due to a lower osmotic potential. The research demonstrates that ABA can regulate stomatal closing to reduce the rate of water loss [38]. Taking all these findings into consideration, we believe that *ScPIP2–1* may have a positive response to PEG 6000 and NaCl stresses through the ABA signal in sugarcane.

What should be stressed here is that we found the overexpression of *ScPIP2–1* in *Arabidopsis* improved its tolerance to salt stress, which was correlated with the higher expression levels of salt-associated marker genes (Fig. 11), lower levels of stress-induced ROS (Fig. 10c), higher activity of ROS scavenging enzyme (Fig. 10d) and higher expression levels of Pro biosynthetic genes and a higher Pro content (Fig. 10e and Figs. 11c, d). In particular, under salt stress, *ScPIP2–1* overexpression *Arabidopsis* displayed healthier physiological states (Fig. 10a), longer length of roots (Fig. 9) and more stable cell ion leakage (Fig. 10b) compared with WT, which suggested that the transgenic *Arabidopsis* had more stable cell pressure. Although the *ScPIP2–1* overexpression improved salt tolerance in *Arabidopsis* in our study, *AtPIP2–1* overexpression resulted in reduced salt stress tolerance in *Arabidopsis* and tobacco [39]. These seemingly conflicting results are not uncommon. In fact, many AQP overexpression studies have produced contrasting results, for example, *AQP* overexpression plants showed either positive or negative effects due to stress tolerance [9, 40]. There are differences in gene regulation, even among highly homologous AQPs, indicating their divergent functions [39]. Another explanation for the contrasting results might lie in the differences in protein sequences between ScPIP2–1 and other plant PIP proteins. Amino acid sequence differences were mainly found in the N-terminus, which was expected to be exposed on the cytosol side [41]. From all the above, it was tempting to infer that contrasting stress phenotypes may be due to differences in the N-termini of the PIP protein (Fig. 3), which contains important motifs such as those for activity regulation, protein stability, protein interaction or even subcellular localization.

In the salt tolerance analysis of transgenic *Arabidopsis*, we found up-regulation of *AtP5CS1* and *AtP5CS2* (Figs. 11c, d), higher Pro content (Fig. 10e) and increased activity of SOD (Fig. 10d) with reduced levels of MDA (Fig. 10c) in *ScPIP2–1* overexpression *Arabidopsis* under salt stress. Pro mainly functions in defense and turgor pressure maintenance against water-deprived conditions [42]. The expression levels of three ion exchange-correlated genes (*AtNHX1*, *AtSOS1* and *AtHKT1*) were up-regulated, which may improve the salt tolerance of *ScPIP2–1* overexpression plants, which is in accordance with a previous report that *AtNHX1*, *AtSOS1* and *AtHKT1* played important roles in salt stress resistance [43]. Hence, the enhanced salt stress tolerance in *ScPIP2–1* overexpression *Arabidopsis* seemed to result in, at least in part, the increased expression of Pro biosynthetic genes and elevated activities of ROS scavenging enzymes, as well as the down-regulation of *AtTRX5* (Fig. 11b). Similar to our findings, the enhanced stress tolerance in *MsPIP2–2* overexpression transgenic *Arabidopsis* was correlated with decreased levels of MDA and increased Pro contents and activity of the SOD enzyme [29]. In the present study, salt tolerance improvement by up-regulation of salt stress-regulated genes and an increase in ROS scavenging enzyme activity suggested that *ScPIP2–1* overexpression might sensitize transgenic *Arabidopsis*, causing the overexpression *Arabidopsis* react faster to salt stress signals and eventually enhance stress defense. According to the results of Figs. 11e and f, which indicated the up-regulation of *AtDREB2* and *AtRD29A* genes under salt stress, we speculate that *ScPIP2–1* in transgenic *Arabidopsis* could activate salt stress sensing or the upstream steps in signaling pathways to induce a better stress-tolerance mechanism than in WT. Consistent with our speculation, it has been suggested that AQPs may be part of an osmotic stress signaling cascade [9]. Additionally, it has even been proposed that AQPs may act as osmosensors [44]. Further study will be required to investigate this possibility.

## Conclusions

In the present study, eight *ScPIP* genes that belong to the *PIP* subfamily of *AQPs* were mined from our previous sugarcane transcriptome database. A full-length cDNA of *ScPIP2–1* was then cloned from ROC22. ScPIP2–1 was localized in the plasma membrane and cytoplasm. The *ScPIP2–1* gene was constitutively expressed in sugarcane tissues, and its transcript levels were increased by ABA, NaCl and PEG 6000 treatments. Yeast two-hybrid screening demonstrated that pBT3-N-*ScPIP2–1* did not possess transcriptional activity and had no toxicity to the yeast strain NMY51. The transient overexpression of *ScPIP2–1* in *N. benthamiana* leaves could enhance the salt tolerance. Moreover, overexpression *ScPIP2–1* in *Arabidopsis* could enhance the salt tolerance of transgenic plants at the phenotypic, physiological and molecular levels. These results would be helpful in mining and functional identification of *ScPIP* family genes in sugarcane in future.

## Methods

### Plant materials and treatments

One panel of sugarcane material was used in this study. The main sugarcane variety ROC22 (*Saccharum* spp. hybrid) in mainland China was provided by the Key Laboratory of Sugarcane Biology and Genetic Breeding, Ministry of Agriculture and Rural Affairs (Fuzhou, China). For tissue-specific expression analysis, 10-month-old ROC22 plants were selected. The samples included the root, bud, + 1 leaf (the youngest fully expanded leaf with a visible dewlap), stem pith and epidermis [45]. For osmotic stress and exogenous hormone treatments, 4-month-old ROC22 plants were cultivated hydroponically for 7 days. The cultivation conditions were 28 °C with 16-h light and 8-h darkness. Then, 25 PEG 6000, 250 mmol·L^− 1^ NaCl and 0.1 mmol·L^− 1^ ABA were used as simulated environmental stress conditions [46–48]. The leaf and root of each treatment were collected at 0, 0.5, 3, 6 and 24 h. Three biological replicates of each sample were prepared, and all the above-mentioned samples were fixed in liquid nitrogen and stored at − 80 °C until total RNA extraction.

### Total RNA extraction and the first strand of cDNA synthesis

Total RNA was extracted using TRIeasy™ Total RNA Extraction Reagent (YEASEN, Shanghai, China), and 1.0 μg total RNA was used to synthesize first-strand cDNA using the Hifair® 1st Strand cDNA Synthesis Kit (gDNA digester plus) (YEASEN, Shanghai, China). The cDNA was detected by 1% gel electrophoresis [49]. The cDNA of 24-h 250 mmol·L^− 1^ NaCl-treated ROC22 leaves was used for gene cloning.

### Identification and bioinformatics analysis of *ScPIP* family genes

Eight *ScPIP* genes were minded from our previous sugarcane transcriptome database [50, 51]. The sequences of these genes were translated and analyzed using the Open Reading Frame Finder (https://www.ncbi.nlm.nih.gov/orffinder/) and DNAMAN V6.0.3.99 software. The signal peptides and *p*Is were predicted using ExPASy (http://us.expasy.org/tools). A phylogenetic tree was constructed with the maximum likelihood (ML) method (1000 bootstrap replicates) using the MEGA X 10.0.5 program [16, 52].

The full-length cDNA sequence of *ScPIP2–1,* which was minded above, was cloned by RT-PCR from 24-h 250 mmol·L^− 1^ NaCl-treated leaf tissue of ROC22. The amplification procedure was set as 94 °C for 5 min, followed by 35 cycles of 94 °C for 30 s, 52 °C for 30 s and 72 °C for 1 min, and then 72 °C for 10 min in an elongation step. The PCR products were purified by a gel-purification kit (Magen, Guangzhou, China), cloned into the pMD 19-T vector (Takara, Dalian, China) and sent for sequencing.

### Subcellular localization assay

The ORF of *ScPIP2–1* without the stop codon was inserted between two restriction sites (*Bam*H I and *Xba* I) of the *35S*::*eGFP* vector (pCAMBIA 2300-*eGFP*). Then, the recombinant vector of *35S*::*ScPIP2–1*::*eGFP* was introduced into *Agrobacterium* GV3101. *Agrobacterium*-mediated transient expression was performed in *N. benthamiana* leaves [53–55]. Subcellular localization of the GFP reporter protein was determined by confocal laser scanning microscopy using a Leica TCS SP5 (Wetzlar, Germany).

### Self-activation of the ScPIP2–1 in yeast

To analyze the transcriptional activity of ScPIP2–1, the DUAL membrane yeast two-hybrid system (including three reporter genes, *HIS3*, *ADE2* and *lacZ*) was used following the manufacturer’s directions for the DUAL yeast two-hybrid system and according to the protocol established in our group [48]. The *ScPIP2–1* gene was PCR-amplified from pMD19-T-*ScPIP2–1* using primers *ScPIP2–1* pBT3-F and *ScPIP2–1* pBT3-R (Table S3). Then, *Sfi* I endonuclease was used to construct the bait vector pBT3-N-*ScPIP2–1*. NMY51 strains carrying the treatment (positive plasmid pBT3-N-*ScPIP2–1*), the negative control (empty vector pBT3-N and empty vector pRR3-N) and the positive control (pNubG-Fe65 and pTSU2-App) vectors were cultured in synthetic dropout medium without leucine and tryptophan (SD/−Leu/−Trp) for 2 days at 30 °C at 150 rpm [48].

In order to verify the self-activation activity of NMY51 strains plus positive plasmid pBT3-N-*ScPIP2–1*, 8.0 μL yeasts plus 1.0 μL X-gal were cultured on solid media, which were SD/−Leu (the synthetic dropout medium without leucine), SD/−Leu/−Trp and SD/−Ade/−His/−Leu/−Trp (the synthetic dropout medium without adenine, histidine, leucine and tryptophan). The sample was diluted tenfold (10^− 1^), one hundred fold (10^− 2^), one thousand fold (10^− 3^) and ten thousand fold (10^− 4^).

### Expression of the *ScPIP2–1* gene in different sugarcane tissues and in response to various abiotic stresses

RT-qPCR analysis was conducted to reveal the expression patterns of *ScPIP2–1* in sugarcane tissues (root, bud, leaf, stem pith and stem epidermis) and in response to various abiotic stresses (NaCl, PEG 6000 and ABA). Glyceraldehyde-3-phosphate dehydrogenase (*GAPDH*, GenBank Accession Number: CA254672) was used as a reference gene (Table S3) [56]. The specific RT-qPCR primers of *ScPIP2–1* (Table S3) were designed using Beacon Designer 8.13 software. An ABI 7500 real-time PCR system (Applied Biosystems, Foster City, CA, USA) and Hieff™ qPCR SYBR Green Master Mix (Low Rox) (YEASEN, Shanghai, China) were used for RT-qPCR analysis. The RT-qPCR reaction system was subjected to 50 °C for 2 min, 95 °C for 10 min, 95 °C for 15 s and 59 °C for 1 min, for 40 cycles. A melting curve analysis was performed at 95 °C for 15 s, 60 °C for 1 min, 95 °C for 15 s and 60 °C for 30 s. Three biological and three technical replicates were performed. The standard curve plot and amplification efficiency of *ScPIP2–1* and *GAPDH* genes in RT-qPCR analysis were showed in Fig. S1 and Table S4. The 2^−∆∆CT^ method [54], SPSS 9 software and Origin 8 software were used to calculate the relative expression of the target gene, to analyze the significance level of the experimental data and to structure the histogram.

### Transient expression of *ScPIP2–1* in *N. benthamiana*

To investigate the function of *ScPIP2–1* under high salt treatment in *N. benthamiana*, the overexpression vector pCAMBIA 1301-*ScPIP2–1* was constructed using T4 ligase (YEASEN, Shanghai, China). The recombinant vector of pCAMBIA 1301-*ScPIP2–1* and the empty vector of pCAMBIA 1301 were separately introduced into *Agrobacterium* GV3101. *Agrobacterium*-mediated transient expression in the *N. benthamiana* leaf was performed [52–54]. The six-leaf stage *N. benthamiana* plants were irrigated with 50 mL of 250 mmol·L^− 1^ NaCl every day. All materials were cultivated at 24 °C with an illumination intensity of 15,000 μmol·m^− 2^·s^− 1^, a photoperiod of 16-h light and 8-h darkness and a relative humidity of 65%. The histocytological analysis was processed by 3, 3′-diaminobenzidine (DAB) staining at 0 h and 24 h on leaves under NaCl stress. At the same time, the chlorophylls and ion leakage of the leaves were determined using soil and plant analyzer development measurements (SPAD, KONICA MINOLTA, Japan) and the soaking method [57], respectively. The expression levels of salt-associated genes, i.e., *NtESI3* (salt stress-induced hydrophobic peptide gene) [58], *NtDREB2* (DREB transcription factor) [59] and *NtP5CS* (correlation Pro production gene) [60] (Table S3), were detected by RT-qPCR. The *NtEF1-α* gene (Table S3) was used as an internal reference for normalization. The standard curve plot and amplification efficiency of RT-qPCR primers were showed in Fig. S1 and Table S4. The 2^−∆∆CT^ method [54], SPSS 9 software and Origin 8 software were used to calculate the relative expression of the target gene, to analyze the significance level of the experimental data and to structure the histogram.

### Stable expression of *ScPIP2–1* in *Arabidopsis* and the salt tolerance analysis of transgenic plants

The *ScPIP2–1* gene was genetically transformed into *Arabidopsis* by *Agrobacterium*-mediated transformation. The overexpression vector was pCAMBIA 1301, which contained a marked gene for HYG resistance. Seeds of transgenic *Arabidopsis* lines of T_1_, T_2_ and T_3_ generations were germinated and selected on 1/2 MS medium with 25 mg·L^− 1^ HYG. As a control, WT seeds were also placed on 1/2 MS medium with HYG. Two weeks later, the seedlings were transferred to soil pots and cultured at 22 °C with an illumination intensity of 15,000 μmol·m^− 2^·s^− 1^, a photoperiod of 16-h light and 8-h darkness and a relative humidity of 65%. The seeds of T_1_, T_2_ and T_3_ generations were generated and collected.

To determine the root lengths, the seeds of WT and three lines of T_3_ transgenic *Arabidopsis* (OE1, OE2 and OE3) were separately placed on 1/2 MS medium containing 0, 50, 150, 200 or 250 mmol·L^− 1^ NaCl. A Nikon camera was used to take the photos 7 days later, and Image J 1.8.0 was used to measure the root lengths [47, 61].

In order to analyze the salt tolerance of *ScPIP2–1* overexpression *Arabidopsis* at physiological and molecular levels, the 5-week plants of WT and three lines of T_3_ transgenic *Arabidopsis* (OE1, OE2 and OE3) were cultivated in peat medium and treated with 50 mL of 250 mmol·L^− 1^ NaCl every day. The Nikon camera was used to take photos at 0, 3 and 7 days post NaCl treatment. The leaf samples for the analysis of physiological indexes were collected at 0 d and 7 d post treatment, and other leaf samples for the analysis of molecular indexes were collected at 0 d, 3 d and 7 d post treatment. Three biological replicates of each sample were prepared. Three plantlets, randomly selected, were defined as one biological replicate, and three biological replicates of each sample were prepared to measure the fresh weight (FW) and dry weight (DW). The RWC was calculated by RWC = (FW-DW) × FW^− 1^ [62]. The contents of MDA and Pro as well as the enzymatic activity of SOD were determined by spectrophotometry using MDA, Pro and SOD assay kits (Solarbio, Beijing, China) [47, 61]. The ion leakage was determined by the soaking method [57]. The expressions of the osmotic stress-defense genes (*AtKIN2*, *AtDREB2* and *AtRD29A*), the ROS-elimination gene (*AtTRX5*), the Pro synthesis-related genes (*AtP5CS1* and *AtP5CS2*) and the ion exchange-related genes (*AtNHX1*, *AtSOS1* and *AtHKT1*) in *Arabidopsis* (Table S3) were analyzed by RT-qPCR [29]. The *AtActin* gene (Table S3) was used as an internal reference for normalization [29]. The standard curve plot and amplification efficiency of these RT-qPCR primers were showed in Fig. S1 and Table S4. The 2^−∆∆CT^ method [54], SPSS 9 software and Origin 8 software were used to calculate the relative expression of the target gene, to analyze the significance level of the experimental data and to structure the histogram.

## Supplementary Information

**Additional file 1: Table S1.** The nucleic acid and amino acid sequences of the *ScPIP* gene family presented in this study.

**Additional file 2: Table S2.** The bioinformatics information of ScPIP proteins.

**Additional file 3: Table S3.** Primers used in this study.

**Additional file 4: Table S4.** The gene amplification efficiency of RT-qPCR in this study.

**Additional file 5: Fig. S1.** The standard curve plot of RT-qPCR primers in this study. Five points of a 10-fold dilution series of cDNA, performed in triplicate wells, amplified using the ABI 7500 real-time PCR system. The standard curve was generated by plotting threshold cycle (Ct) values against relative input cDNA dilution ratio. Taking logarithm value of cDNA dilution ratio as X-axis, ΔCt of gene as Y-axis. The cDNA of ROC22 leaf tissue was used for the standard curve analysis of the *ScPIP2–1* and *ScGAPDH* genes. The cDNA of wild-type *Arabidopsis* Col-0 leaf tissue was used for the standard curve analysis of the *AtKIN2*, *AtTRX5*, *AtP5CS1*, *AtP5CS2*, *AtDREB2*, *AtRD29A*, *AtNHX1*, *AtHKT1*, *AtSOS1* and *AtACT* genes.

## Data Availability

The datasets supporting the conclusions of this article are available in the NCBI repository (https://www.ncbi.nlm.nih.gov/) with the GenBank accession numbers of MZ362000, MZ362001, MZ362002, MZ362003, MZ362004, MZ362005, MZ362006 and MZ362007. This is also to confirm that we have got the administrative permission to access and use the sugarcane transcriptome database reported by Zhou et al. [50] and Yang et al. [51].

## Abbreviations

AQPs: Aquaporins
MIP: Major intrinsic protein
PIPs: Plasma membrane intrinsic proteins
TIPs: Tonoplast membrane intrinsic proteins
NIPs: Nodulin26-like major intrinsic proteins
SIPs: Small and basic intrinsic proteins
XIPs: X intrinsic proteins
PEG: Polyethylene glycol
NaCl: Sodium chloride
ABA: Abscisic acid
ROS: Reactive oxygen species
RT-PCR: Reverse transcription-polymerase chain reaction
ORF: Open reading frame
*p*I: Isoelectric point
RT-qPCR: Real time quantitative PCR
RWC: Relative water content
Pro: Proline
MDA: Malondialdehyde
SOD: Superoxide dismutase
HYG: Hygromycin
FW: Fresh weight
DW: Dry weight
GAPDH: Glyceraldehyde-3-phosphate dehydrogenase
