# Supplementary figures and images for: A PIP-mediated osmotic stress signaling cascade plays a positive role in the salt tolerance of sugarcane
Source: BMC Plant Biol. 2021 Dec 13;21:589. doi: 10.1186/s12870-021-03369-9 (PMC8667355; doi:10.1186/s12870-021-03369-9)

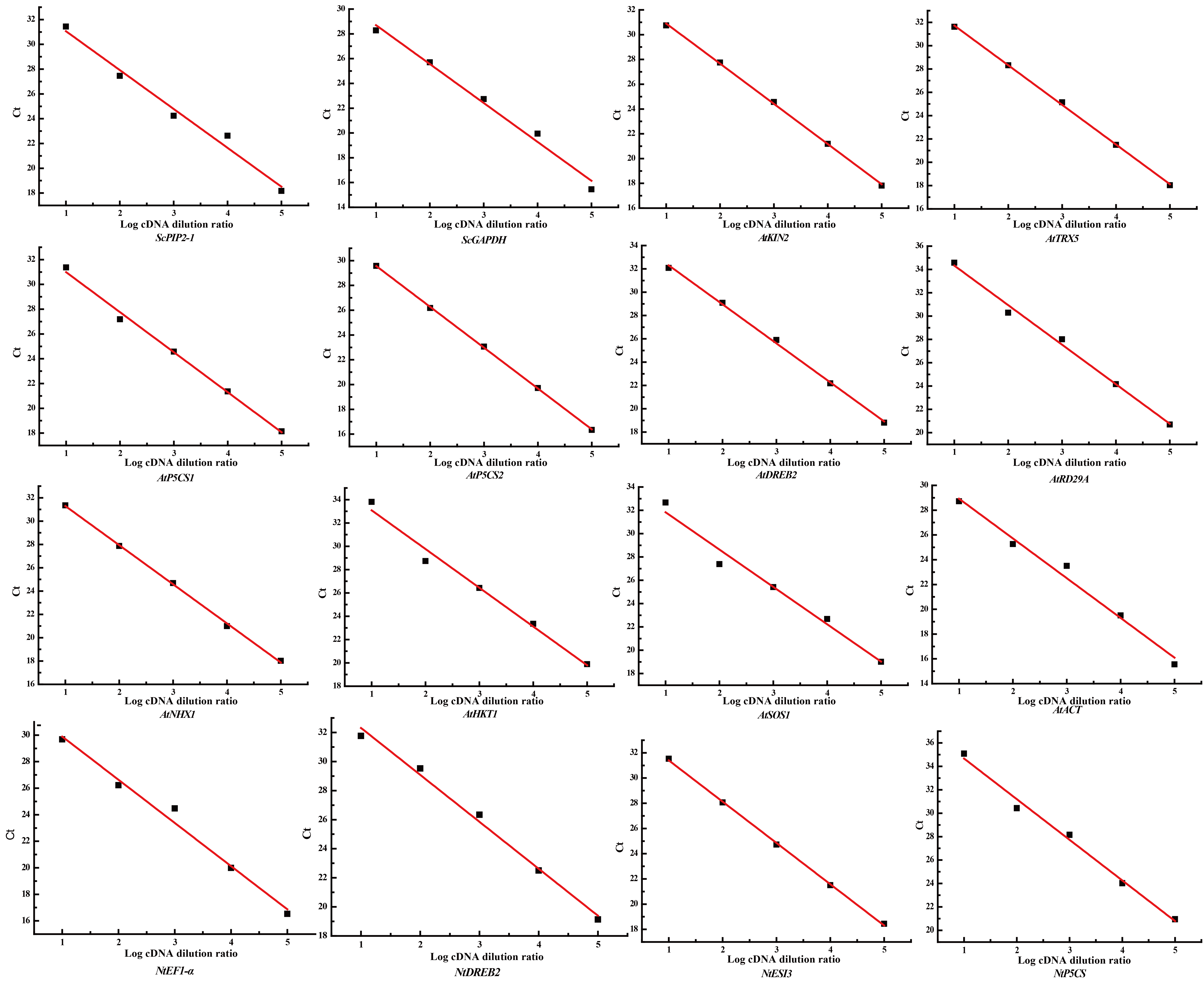

Supplement: Supplementary file 5 — Additional file 5: Fig. S1. The standard curve plot of RT-qPCR primers in this study. Five points of a 10-fold dilution series of cDNA, performed in triplicate wells, amplified using the ABI 7500 real-time PCR system. The standard curve was generated by plotting threshold cycle (Ct) values against relative input cDNA dilution ratio. Taking logarithm value of cDNA dilution ratio as X-axis, ΔCt of gene as Y-axis. The cDNA of ROC22 leaf tissue was used for the standard curve analysis of the ScPIP2–1 and ScGAPDH genes. The cDNA of wild-type Arabidopsis Col-0 leaf tissue was used for the standard curve analysis of the AtKIN2, AtTRX5, AtP5CS1, AtP5CS2, AtDREB2, AtRD29A, AtNHX1, AtHKT1, AtSOS1 and AtACT genes. [file 12870_2021_3369_MOESM5_ESM.tif]
